# Risk Assessment Tools and Data-Driven Approaches for Predicting and Preventing Suicidal Behavior

**DOI:** 10.3389/fpsyt.2019.00036

**Published:** 2019-02-13

**Authors:** Sumithra Velupillai, Gergö Hadlaczky, Enrique Baca-Garcia, Genevieve M. Gorrell, Nomi Werbeloff, Dong Nguyen, Rashmi Patel, Daniel Leightley, Johnny Downs, Matthew Hotopf, Rina Dutta

**Affiliations:** ^1^Institute of Psychiatry, Psychology and Neuroscience, King's College London, London, United Kingdom; ^2^School of Electrical Engineering and Computer Science, KTH Royal Institute of Technology, Stockholm, Sweden; ^3^South London and Maudsley NHS Foundation Trust, London, United Kingdom; ^4^National Center for Suicide Research and Prevention (NASP), Department of Learning, Informatics, Management and Ethics (LIME), Karolinska Institutet, Stockholm, Sweden; ^5^National Center for Suicide Research and Prevention (NASP), Centre for Health Economics, Informatics and Health Services Research (CHIS), Stockholm Health Care Services (SLSO), Stockholm, Sweden; ^6^Department of Psychiatry, IIS-Jimenez Diaz Foundation, Madrid, Spain; ^7^Department of Psychiatry, Autonoma University, Madrid, Spain; ^8^Department of Psychiatry, General Hospital of Villalba, Madrid, Spain; ^9^CIBERSAM, Carlos III Institute of Health, Madrid, Spain; ^10^Department of Psychiatry, University Hospital Rey Juan Carlos, Móstoles, Spain; ^11^Department of Psychiatry, University Hospital Infanta Elena, Valdemoro, Spain; ^12^Department of Psychiatry, Universidad Católica del Maule, Talca, Chile; ^13^Department of Computer Science, University of Sheffield, Sheffield, United Kingdom; ^14^Division of Psychiatry, University College London, London, United Kingdom; ^15^Alan Turing Institute, London, United Kingdom; ^16^School of Informatics, University of Edinburgh, Edinburgh, United Kingdom

**Keywords:** suicide risk prediction, suicidality, suicide risk assessment, clinical informatics, machine learning, natural language processing

## Abstract

Risk assessment of suicidal behavior is a time-consuming but notoriously inaccurate activity for mental health services globally. In the last 50 years a large number of tools have been designed for suicide risk assessment, and tested in a wide variety of populations, but studies show that these tools suffer from low positive predictive values. More recently, advances in research fields such as machine learning and natural language processing applied on large datasets have shown promising results for health care, and may enable an important shift in advancing precision medicine. In this conceptual review, we discuss established risk assessment tools and examples of novel data-driven approaches that have been used for identification of suicidal behavior and risk. We provide a perspective on the strengths and weaknesses of these applications to mental health-related data, and suggest research directions to enable improvement in clinical practice.

## Targeted Suicide Prevention–Time for Change?

Suicide is a global public health concern, with more than 800,000 worldwide deaths, annually. The World Health Organization has set a global target to reduce the rates of suicides by 10% by 2020 (1, 2) The concept of suicidal behavior encapsulates thoughts, plans and acts an individual makes toward intentionally ending their own life (3).

For targeted suicide prevention strategies to be effective for those with mental health problems, high-quality and accessible data from health services is essential (1). With the increased availability of electronic data from public health services and patient-generated data online, advances in data-driven methods could transform the ways in which psychiatric health services are provided (4–6).

Here, we discuss and contrast the use of risk assessment tools and data-driven computational methods, such as the use of machine learning and Natural Language Processing (NLP) within the precision medicine paradigm to aid individualized care in psychiatry. Our aim is to convey the strengths, but also the limitations of current approaches, and to highlight directions for research in this area to move toward impact in clinical practice.

## Risk Assessment Tools for Suicide Prediction and Prevention

Tools developed for suicide risk assessment include psychological scales, e.g., the Beck Suicide Intent Scale, and scales derived from statistical models, e.g., the Repeated Episodes of Self-Harm score. Completing suicide risk assessments have become a mandatory part of clinical practice in psychiatry (7), absorbing a considerable proportion of time allocated to clinical care (8). Assessments are aimed at identifying treatable and modifiable factors, the premise being that identifying “high” suicide risk allows clinicians to enhance service provision, or to implement suicide prevention measures in specific, “high-risk” patient-groups, thus avoiding the implementation of inappropriate or costly interventions in “low-risk” patients.

Recently published meta-analyses suggest that the existing tools have inadequate reliability and low positive predictive value (PPV) in distinguishing between low and high-risk patients (9–13). For instance, the meta-analytically derived PPV of studies based on 53 samples was 5.5% over an average follow-up period of 63 months (12). The majority of suicides occurred in the patient groups categorized as “low-risk,” as they vastly outnumber the “high-risk” group. Furthermore, most patients in the “high-risk” group did not die by suicide, because of the relative rarity of the outcome. In addition, the 5.5% risk of suicide for “high-risk” patients pertains to a time interval of more than 5 years; from a clinical perspective it is more helpful to identify those likely to die by suicide within much shorter time frames, namely weeks or months. No relationship between the precision of risk assessment tools and date of their publication has been found, suggesting no radical improvement over the past 50 years (10, 12).

Suicide is a rare outcome, even in individuals with severe mental health disorders. This presents several challenges when it comes to suicide risk assessment. Low base-rates demand an instrument with very strong predictive validity. The performance of almost all instruments fall short of this—a mean sensitivity of 56% and a specificity of 79% was derived from a recent meta-analysis (12). But, even a detection method with 90% sensitivity and 90% specificity, would still lead to low PPV-5% at a 1 year incidence of 500/100,000 in clinical populations at higher risk (14). In addition, a time interval of 1 year is not useful in practice. If the goal of prediction was to identify suicide risk within a fortnight or a month, these risk tools would offer little extra predictive power above chance.

Current methods of building suicide risk assessments stem from translating clinical observations, and theory, into either fixed scales or risk factors to inform statistical models. It is possible that these approaches have reached their limits, since there has been no improvement over several decades, and new scales are costly to implement within already stretched front-line services. Suicidal behavior is a complex phenomenon which is contextually dependent and can shift rapidly from 1 day to the next. Capturing these dynamics requires sophisticated measurement and statistical models (15).

One promising direction is to make use of information generated routinely over the course of everyday public service and research activity that deliver dynamic risk assessment at the point of care. This real-world data (RWD) can come from sources such as case reports, administrative and healthcare claims, electronic health records (EHRs), or public health investigation data. RWD show promise in generating new, previously unknown hypotheses with data-driven machine learning techniques, e.g., detection of previously unknown risk or mitigating factors, adverse effects or treatments for mental health disorders (16, 17).

## Data-Driven Approaches

Machine learning techniques are methods that learn from and model large datasets using statistical and algorithmical approaches. They can be used to model risk factors, patterns of illness evolution and outcomes, on a speed and scale that is impossible for humans. These models use features to provide information on future events, such as the likelihood that a patient will attempt suicide within a given time interval, and can model complex relations between features and outcomes. Clinical databases such as EHRs typically contain a variety of data, of which structured data entries lend themselves well to computational analysis. As an alternative, or complementary approach to risk assessment tools, data mining techniques have been applied to the problem of identifying suicidal behavior and assessing suicide risk, using different levels of detail and cohorts (18–24), examples in Table 1. These findings indicate that machine learning approaches applied to RWD have potential and could be used to generate tools to improve e.g., medical decision-making and patient outcomes. Owing to the flexibility of these approaches, the models can be continuously updated to refine and improve their clinical applicability.

**Table 1:** Six example studies published between 2014 and 2017 that use data-driven approaches—machine learning and/or natural language processing (NLP)-for classifying or predicting suicide risk.

**References**	**Task**	**Data source; Approach**	**Key results and findings**
Barak-Corren et al. (19)	Prediction of patients' future risk of suicidal behavior	Partners Healthcare Research Patient Data Registry, US EHR 1998–2012; Bayesian machine learning	33–45% sensitivity, 90–95% specificity, and early (3–4 years in advance on average). The approach identified well-known risk factors (e.g., substance abuse) but also less conventional risk factors (e.g., certain injuries and chronic conditions)
Kessler et al. (22)	Prediction of suicides after psychiatric hospitalization	HADS: data from 38 Army/DoD administrative data systems, US; elastic net (regression trees, penalized regressions)	Higher risk of suicide within 12 months of hospital discharge compared to total Army. Strongest predictors included socio-demographics (male, late age of enlistment), criminal offenses (verbal violence, weapons possession), prior suicidality, aspects of prior psychiatric inpatient and outpatient treatment, and disorders diagnosed during the focal hospitalizations
McCoy et al. (25)	Prediction of suicide and accidental death after discharge	Massachusetts General Hospital and Brigham and Women's Hospital, Boston, US EHRs; NLP approach to characterize positive and negative valence (compared with model using only structured codes)	Positive valence reflected in narrative notes was associated with a 30% reduction in risk for suicide
Metzger et al. (26)	Epidemiological surveillance of suicide attempts	Lyon University Hospital Emergency Department, France; Random forest and naïve Bayes including NLP derived variables	Automatic detection of suicide attempts ranged from 70.4 to 95.3% F-measure. Improved quality of epidemiological indicators as compared to current national surveillance approaches.
Tran et al. (23)	Risk stratification using EHR data, compared with clinician assessments	Barwon Health, Australia, EHRs from inpatient admissions and ED visits; L1-penalized continuation-ratio model for ordinal outcomes	Clinicians using checklist predicted patients at high-risk in 3 months with AUC 0.58, 95% CIs: 0.50–0.66. The data-driven model was superior: AUC 0.79, 95% CIs: 0.72–0.84. Predictive factors included known risks for suicide, but also other information relating to general health and health service utilization
Walsh et al. (24)	Prediction risk of suicide attempt	Vanderbilt University Medical Center, US, BioVU Synthetic Derivative data repository; Random forest	Future suicide attempts were predicted with AUC 0.84, precision 0.79, recall 0.95, Brier score 0.14. Accuracy improved from 720 days to 7 days before the suicide attempt. Predictor importance shifted across time.

## Free-text and Natural Language Processing

One main advantage with EHR data is that it captures routine clinical practice, which may hold cues for suicidal behavior amongst individuals in contact with health services. Detailed clinical information in EHRs is predominantly recorded in free text fields (e.g., clinical case notes and correspondence). Text records contain rich descriptive narratives—describing symptoms, behaviors and changes experienced by patients, which are elicited during clinical assessment and follow-up (27). Criterion-based classification systems (e.g., ICD-10 and DSM-5) do not necessarily reflect the underlying etiology and pathophysiology at an individual patient level (28), and genetic and environmental risk factors are shared between different mental disorders (29). Thus, a richer and more reliable picture of what is documented in EHRs needs to include an analysis of the textual content, which is where NLP methods are important.

Recent years have seen an increase in use of NLP and text mining tools to extract clinically relevant information from EHR and other biomedical text (30–33). Information extraction is an established subfield within NLP seeking to automatically derive structured information from text. In the mental health domain, NLP has been used to extract and classify clinical constructs such as symptoms, clinical treatments and behavioral risk factors (34–41). Using NLP approaches to identify patients at risk of suicidal behavior in addition to, or in combination with, structured data can increase both precision and coverage (26, 42–45).

Other text-based aspects can also be important to the full understanding of suicide risk. For instance, frequent use of third-person pronouns in EHRs, indicating interpersonal distance, has been found to be discriminative for patients who died from suicide, with an increased relative frequency closer to the event (46). Positive valence in discharge summaries (e.g., terms like *glad, pleasant*) has also been associated with diminished risk of death by suicide (25).

## Looking Ahead: the Role of Data-Driven Approaches

The distinctive advantage of data-driven approaches is that they may be powerful even if the PPV of the predictions are low, because they can be deployed on a large scale. The usefulness is dependent on the cost and efficacy of the possible intervention. If an automated model reduced the suicide risk by just a fraction, it could save numerous lives cost-efficiently. If we accept that investment in machine learning and NLP approaches is needed to improve predictive and preventive measures for identifying suicide risk (10), focus should now be placed to make these methods applicable in clinical reality (6, 47).

### Obtaining and Utilizing Quality Data

The success of machine learning and NLP approaches depends on several factors, such as data availability and task difficulty. EHRs are not easily shared due to confidentiality and governance constraints, thus method comparison, reliability analysis and generalizability studies are still uncommon. Suicidality represents a broad spectrum of actions and thought processes. There is a wide range of clinical practice in labeling suicide-related phenomena within and across nations. With researchers struggling to settle on standardized nomenclature on non-fatal suicidal behaviors and uniformity in classifying “ideation,” this presents considerable challenges to devise an inclusive but specific framework for using NLP to extract relevant material from text sources (e.g., defining appropriate suicide-related keywords). In order to gather a sufficient number of terms, a keyword search strategy on an entire EHR database is commonly used. Whilst effective for unambiguous concepts such as “anemia” or “migraine,” this may result in an artificially simplified sample where synonymous terms are missed. For example, from a manually reviewed small EHR sample, suicidal ideation was expressed with alternative phrases such as “go to sleep and not wake up” or “jump off a bridge.” However, generating high quality data is time-consuming and costly. Applying keyword matching methods on a large data sample may still result in high coverage (48). Methods to iteratively refine and extend appropriate keywords and data samples for generating high quality annotations on text data can help minimize development costs.

### How Can Data-Driven Models be Explained?

While the effectiveness of data-driven approaches has been increasing rapidly due to both technological advancements (e.g., in deep neural networks) and the availability of larger and richer datasets, many approaches are overly opaque. The underlying prediction models are developed on large, complex datasets with a multitude of features and data points that are internally condensed into abstracted representations which are difficult for humans to interpret. Acceptance of data-driven risk prediction models by healthcare practitioners and patients, involves ensuring that the model output can be clinically trusted (49). The increasing interest in algorithmic accountability (50) is thus a welcome development. For example, in a project on evaluating the use of machine learning methods to predict the probability of death for pneumonia patients, neural network models were most accurate, but discarded in favor of simpler models, because they were more intelligible (49). Advanced machine learning methods rely on numerous parameters and configurations, which need to be made interpretable and understandable in order to support practitioners in judging the quality of the assessment, and help identify confounding factors in the decision process.

For example, a suicide risk model developed with an advanced machine learning approach using large numbers of features from EHRs, such as symptoms and behavioral patterns, will produce a model that outputs a risk score but without an explanation of how the score was derived. Making machine learning models comprehensible could be done in different ways (51). One alternative is to extract a more interpretable model, e.g., decision trees, from an underlying “black-box” model (52) by for instance visualizing the most important features and providing an interface to analyse these. Another approach could be to explain a particular predictive outcome rather than explaining the complete model (53), or by visualizing the strength of different model weights and features as in recent text applications (54–56). Further, recent advances in developing patient similarity models could be a valuable approach to develop visual representations and models for improved outcome prediction (57).

The concept of interpretability is not well-defined (58) and there is as yet no consensus on how to evaluate the *quality* of an explanation (59). Explanations should be tailored toward the specific task and the end users; employing and testing the output scores and explanations in a practical setting (60).

### Toward Impact in Clinical Practice

Although recent studies using data-driven methods show promising results, there is still much more work to be done to improve their predictive *utility*, even within “high-risk” cohorts such as those who actually reach health care services. NLP and machine learning methods are still far from perfect. The need to account for the *longitudinal* nature of EHRs is challenging—e.g., establishing a pattern of behavior or treatment response where symptoms may fluctuate over time (61). *Changes* in symptoms, behaviors and healthcare service use prior to suicidality are often strong predictors and need to be appropriately modeled.

The main advantage with data-driven approaches compared to time-consuming risk assessment tools is that they can be continuously refined and updated, they are bespoke, and the data is already there. Access to computing power and data no longer requires huge investment (62, 63). An example of a decision support tool that would support a clinician in their daily work could be one that automatically generates a summary based on a patient's previous history, compared with a larger population trajectory. The tool could output a risk score, highlight which data elements were used to infer this score, and provide the clinician support to conduct further interactive analyses.

However, the main limiting factor for progress in deploying these types of models in clinical practice lies in the lack of clarity around data governance standards and large-scale solutions for patient consent, particularly cross-institutionally. Further, these methodological advances are fairly recent compared to risk assessment tools, and are still continuously being developed. Support for interdisciplinary environments where technical expertise alongside clinical is necessary to enable validation and deployment into clinical practice.

**Figure 1:**
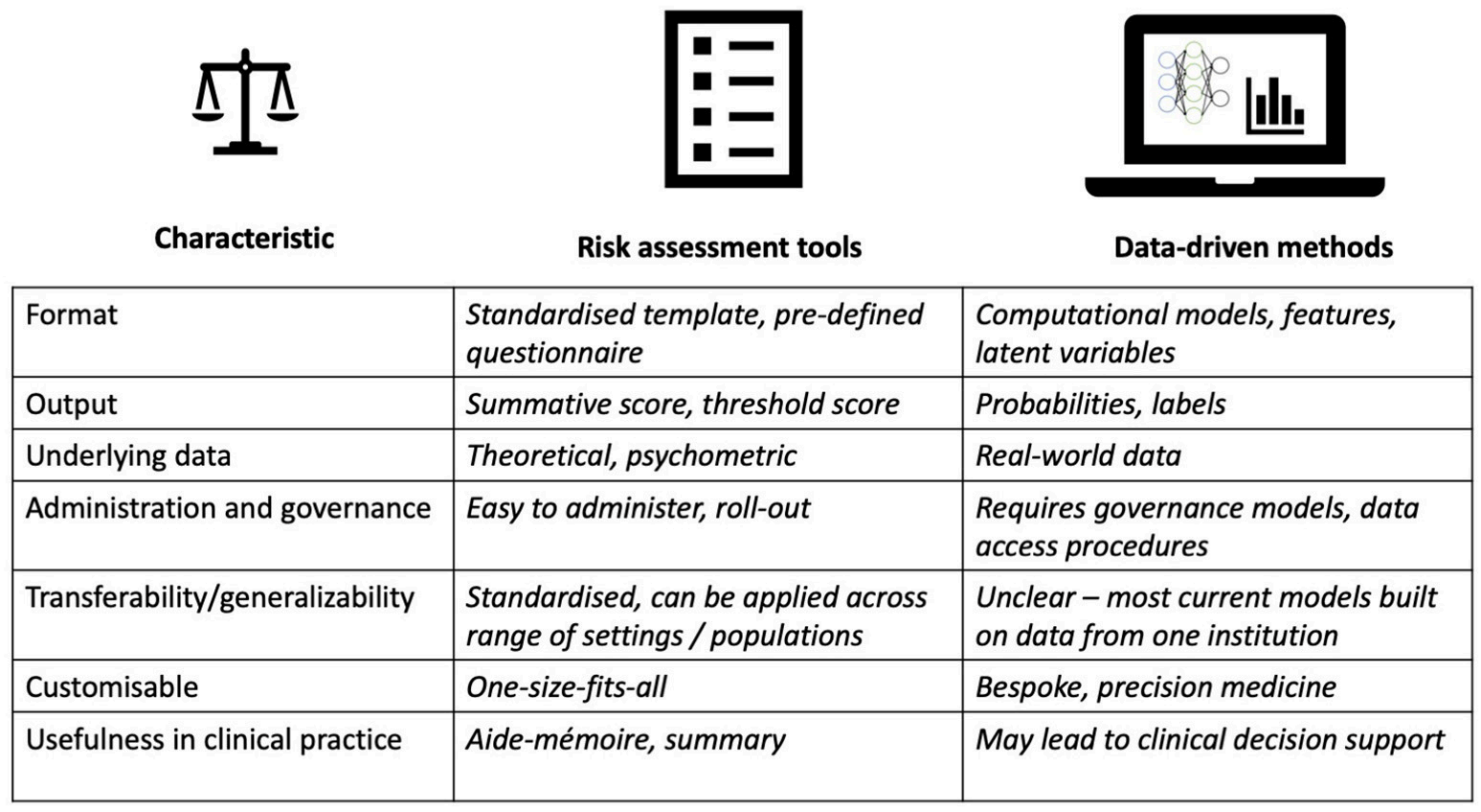
Summary of main characteristics (left) underlying suicide prediction and prevention models: format, output, underlying data, administration and governance, transferability/generalizability, customizable, usefulness in clinical practice. Risk assessment tools (middle) compared with data-driven models (right).

Preventing suicides on a national or even international scale requires multiple societal and health care service considerations (64, 65). To incorporate new technological support that may aid clinicians in their daily work, data-driven methods need to be developed in a way that they actually provide actionable and interpretable information. The main advantages of risk assessment tools in clinical practice are that they are standardized, easy to administer, learn, and interpret—but because they offer little or no predictive ability, they could be enhanced, adapted or complemented by data-driven models that better reflect the individual patient situation (Figure 1).

### Beyond the Clinic

With the increased use of social media, there is a growing source of text online related to mental health, including suicidal behavior, that can be analyzed with data-driven methods (66–68). The growth of online support networks is an issue that could be integrated in research and health and social care processes (69). Deploying prevention systems that can also operate to improve public health and wellbeing is a another area of growing interest to researchers and policy makers (70, 71). A considerable number of suicides occur in people who have not received any prior mental health assessment or treatment (72). Reliable suicide detection from data generated outside of the healthcare setting is one way of addressing this issue. For instance, moderated online social media-based therapy has been successfully developed for first episode psychosis patients (73). With appropriate ethical research protocols in place (74), this approach could serve as inspiration for developing moderated intervention programmes open to the public based on retrospective large-scale, diverse non-clinical data sources.

## Conclusions

Over the last decade there has been an important shift in medical care, with an active role for patients in their care. Clinicians are encouraged to sustain a reciprocal and collaborative relationship with their patients; enshrined in the 4Ps-predictive, preventive, personalized and participatory medicine (75). The ubiquity of IT technology, increase in education level, and maturation of digital natives have all contributed to an active role for patients. In fact, in 2013, 24% of adults in Europe were millennials aged 18–33 (76). Researchers need to be sensitive to not just the engagement of patients but also the ethical issues of using IT in novel strategies with potential patient benefit (5), so avoiding the public concern and mistrust which followed the introduction of *care.data* in England (77), and recent events with Cambridge Analytica and Facebook.

Today, we are in a unique position to utilize a vast variety of data sources and computational methods to advance the field of suicide research. To address the inherent complexity of suicide risk prediction, collaborative, interdisciplinary research environments that combine relevant knowledge and expertise are essential to ensure that the requisite clinical problem is addressed, that appropriate computational approaches are employed, and that ethical considerations are integrated in the research process when moving toward participatory developments.

## Author Contributions

SV and RD proposed the manuscript and its contents. All authors participated in the workshop The Interplay of Evaluating Information Extraction approaches and real-world Clinical Research that was held at the Institute of Psychiatry, Psychology and Neuroscience, King's College London, April 27 2017, and financially supported by the European Science Foundation (ESF) Research Networking Programme Evaluating Information Access Systems: http://elias-network.eu/. SV and RD outlined the first draft of the manuscript. Each author contributed specifically to certain manuscript sections: GH on risk assessment tools, EB-G on data-driven methods, GG on NLP, NW, JD, and RP on NLP specifically for mental health, DN on explainability of data-driven methods, DL on deployment and real-world implications, MH on the overall manuscript. All authors contributed to editing and revising the manuscript. SV incorporated edits of the other authors. All authors approved the final version.

### Conflict of Interest Statement

The authors declare that the research was conducted in the absence of any commercial or financial relationships that could be construed as a potential conflict of interest.
